# Rapid identification of inflorescence type markers by genotyping-by-sequencing of diploid and triploid F_1_ plants of *Hydrangea macrophylla*

**DOI:** 10.1186/s12863-019-0764-6

**Published:** 2019-07-23

**Authors:** Conny Tränkner, Jörg Krüger, Stefan Wanke, Julia Naumann, Torsten Wenke, Frauke Engel

**Affiliations:** 1 Leibniz Institute of Vegetable and Ornamental Crops, Kühnhäuser Straße 101, 99090 Erfurt, Germany; 2 Institut für Botanik, Technische Universität Dresden, Zellescher Weg 20b, 01062 Dresden, Germany; 3 ASGEN GmbH & Co. KG, Egon-Erwin-Kisch-Str. 6, 01069 Dresden, Germany; 4 Gartenbau Kötterheinrich Hortensienkulturen, Hohner Mark 20, 49525 Lengerich, Germany; 5 Present Address: Erfurt Research Centre for Horticultural Crops, Erfurt University of Applied Sciences, Kühnhäuser Straße 101, 99090 Erfurt, Germany

**Keywords:** Genotyping-by-sequencing, Polyploidy, Unreduced gametes, Breeding, Marker associated selection, Ornamental

## Abstract

**Background:**

The ornamental crop *Hydrangea macrophylla* develops highly attractive lacecap (wild type) or mophead inflorescences. The mophead trait, which is mostly favored by consumers, is recessively inherited by the *INFLORESCENCE TYPE* locus (*INF*). If lacecap cultivars are crossed with mophead cultivars, then either 50% or all progenies develop lacecap inflorescences, depending on the zygosity at the *INF* locus. For most cultivars, the zygosity at the *INF* locus is unknown. Furthermore, the determination of the inflorescence type in offspring populations is time-consuming, because seedlings flower the first time in the 2nd year after sowing. Within this study, we aimed to develop DNA-based markers that allow to determine the zygosity at the *INF* locus of prospective parental plants and to predict the inflorescence phenotype of seedlings already in the non-flowering stage.

**Results:**

By crossing a mophead and a lacecap cultivar of *H. macrophylla*, we produced a pseudo-backcross F_1_ population consisting of 422 plants. These plants segregated into 279 lacecap, 73 mophead, 3 intermediate and 67 non-flowering plants, differing significantly from the expected 1:1 segregation ratio. Surprisingly, 75% of these plants were triploid, although both parents were diploid. We found that the lacecap parent produced unreduced pollen, which induced the formation of triploids. 380 randomly selected F_1_ plants were genotyped by genotyping-by-sequencing (GbS). Using a genome assembly of cultivar ‘Sir Joseph Banks’, we performed subsequently a bulk sequence analysis with pooled GbS data of diploid versus mophead plants. We identified directly 2 markers tightly linked with the *INF* locus, each of them explaining 99.7% of the inflorescence phenotype. Using a collection consisting of 56 diploid, triploid or tetraploid *H. macrophylla* varieties, we detected 6 sequence variants for one of these markers. Two variants were associated with the mophead phenotype. Furthermore, we found by marker analysis a co-segregation between the mophead and the non-flowering trait, which indicates a major flowering time locus next to the *INF* locus.

**Conclusion:**

Through bulk sequence analysis of pooled GbS data from diploid and polyploid F_1_ plants, we identify rapidly tightly linked markers for the inflorescence type, a dominant-recessively inherited trait in the non-model plant species *H. macrophylla*.

**Electronic supplementary material:**

The online version of this article (10.1186/s12863-019-0764-6) contains supplementary material, which is available to authorized users.

## Background

*Hydrangea macrophylla* (Thunb.) Ser. belongs to the genus Hydrangea, family Hydrangeaceae. Recent molecular analyses have grouped 208 species into this genus [1]. These species are native to East and Southeast Asia and to the Americas. Especially the Asian species have been used for breeding and the resulting cultivars are well-known ornamentals, which belong to the upmarket segment of ornamentals. Out of these, *H. macrophylla* is the economical most important one, because of its attractive foliage and its impressive floweriness in form of many large, colorful inflorescences. Cultivars of *H. macrophylla* are used for landscaping in all temperate regions around the world, as potted plants for indoor growing or for production of fresh or dry cut flowers.

**Fig. 1:**
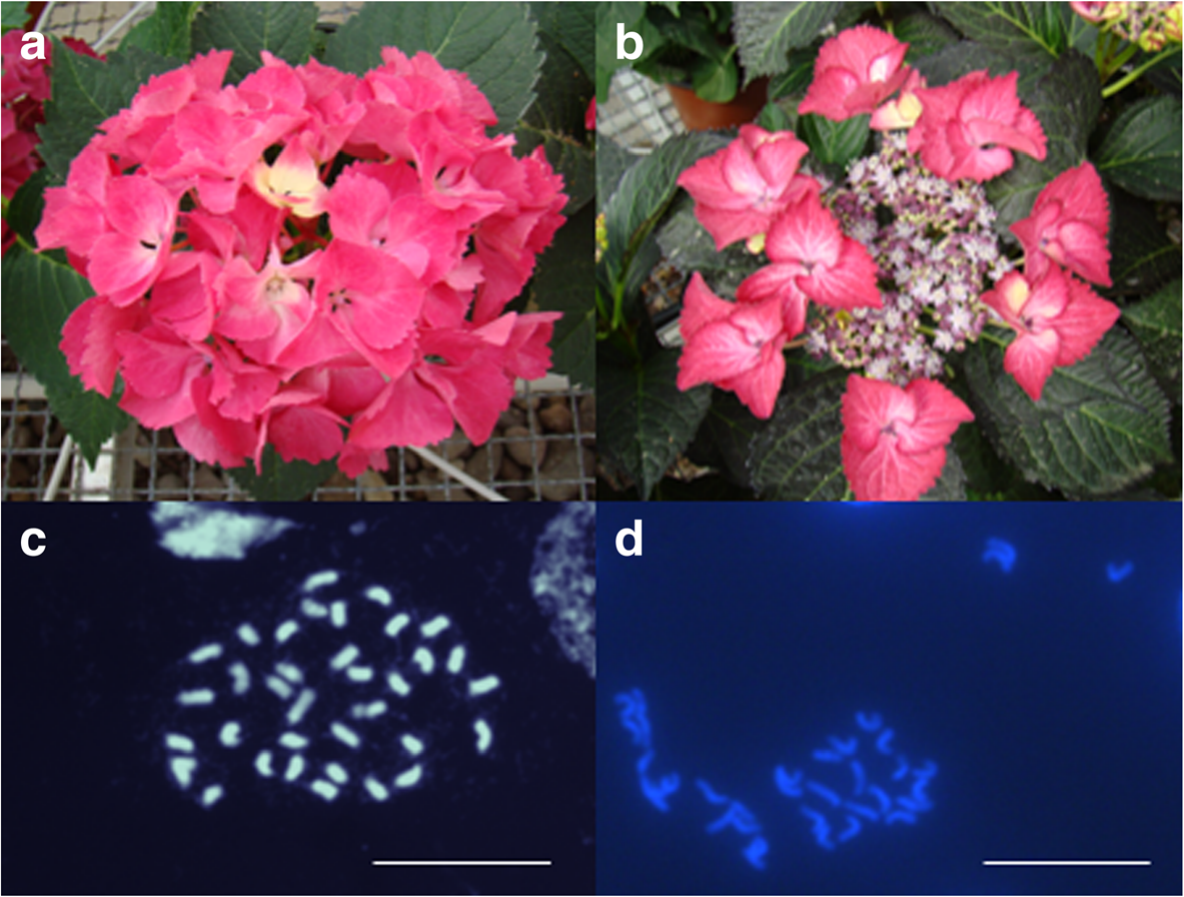
Inflorescences and chromosomes of the parental plants that produced after crossing mophead and lacecap, diploid and triploid F_1_ progenies. The mophead (**a**, **c**) as well as the lacecap (**b**, **d**) parent possess *2n* = *2x* = 36 chromosomes. Bar = 20 μm

Inflorescences of *H. macrophylla* consist of decorative and non-decorative flowers. The decorative flowers appear large due to white, pink, purple, red or blue colored showy sepals, whereas non-decorative flowers seem to be smaller due to inconspicuous, green sepals. Furthermore, decorative and non-decorative flowers differ in the number of floral organs, although petals, stamens and pistils look similar in both types of flowers. Both flower types show male and female fertility [2]. These flowers are clustered in cymose corymbs forming either lacecap or mophead (hortensia) inflorescences. Lacecap inflorescences refer to the wild type. This inflorescence type consists of many non-decorative flowers located in the center and decorative flowers located at the periphery of the inflorescence. All flowers form a flat corymb (Fig. 1b). In contrast, mophead inflorescences are convex, have often fewer flowers than lacecap inflorescences and contain a higher proportion of decorative flowers, which are distributed across the inflorescence resulting in a hemisphere or even a whole sphere form (Fig. 1a). While the inflorescence architecture differs regarding the morphology of pedicels, the number of secondary inflorescences on the axis of the primary inflorescence and the positions of decorative and non-decorative flowers, the seasonal development of both of these inflorescence types is similar [3]. Spontaneous somatic mutations can alter the inflorescence type from lacecap to mophead and also from mophead to lacecap [4].

Consumers prefer mophead cultivars, due to the ornamental value of the dominating decorative flowers. Thus, the mophead inflorescence type is under breeder’s consideration. Mostly, the method of clone breeding is applied for *H. macrophylla*, due to widespread self-incompatibility in *Hydrangea* and the vegetative propagation via cuttings in *Hydrangea* production systems. In clone breeding, selection occurs already in the F_1_ generation, followed by vegetative propagation of selected F_1_ plants. Uemachi and Okumura [4] showed that the inflorescence type is genetically controlled by a single major locus in a dominant-recessive manner, in which the mophead type is inherited recessively and the lacecap type dominantly. If crosses are performed between mophead and lacecap varieties, then all progenies will be either lacecap or segregate in a ratio of 1:1 in lacecap and mophead plants. In complex breeding programs, the mophead type is maintained by (pseudo-)backcrosses or by crossing heterozygous half or full siblings, which result in segregation ratios of 1:1 or 3:1 for lacecap and mophead. The inflorescence type of seedlings can be recorded earliest in the 2nd year after sowing, because seedlings need at least 13 months to develop flowers. Depending on the crossing parents, about 50, 75 or even 100% lacecap seedlings are cultivated, before they can be identified and discarded if only mophead plants are desired. Here, DNA-based markers would allow the selection of mophead progenies already in the year of sowing, which significantly reduces space, labor and costs.

The development of markers is mostly based on mapping populations. Due to the widespread self-incompatibility of *H. macrophylla*, informative mapping populations will result mostly from crosses between two heterogeneous parents. The corresponding progenies will be strongly heterozygous resulting in a strong variation at genotypic and phenotypic level. *H. macrophylla* has a basis number of 18 chromosomes with a genome size of about 2.2 Gbp. However, diploid and triploid cultivars exist, which cannot be distinguished by eye. Diploid cultivars have 36 chromosomes with 2C DNA contents ranging from 3.85 to 4.97 pg, whereas triploid cultivars have 54 chromosomes with 2C DNA contents from 6.48 to 7.27 pg [5–11]. Occasionally, crosses between diploids result in diploid and triploid plants, suggesting the genotype-specific production of unreduced gametes [10, 12]. Recently, Waki et al. [13] published the first genetic map of *H. macrophylla* based on 93 F_2_ plants and 226 simple sequence repeat (SSR) markers. Using the inflorescence type as phenotypic dominant marker, they mapped the *INF* locus (named *h*_*frau*_) to linkage group 4 and identified through detailed linkage analysis the flanking markers HS527 and HS071 that explained 93.5 and 96.3% of the inflorescence phenotype, respectively.

In the presented study, we analyzed a F_1_ population of *H. macrophylla*, which segregated into diploid as well as triploid mophead and lacecap plants. Partial genome-wide sequencing of these F_1_ plants was performed through genotyping-by-sequencing. This method uses methylation-sensitive restriction enzymes to reduce the complexity of genomic DNA and targets only on lower copy regions across the genome [14]. We developed a pipeline for bulk sequence analysis of pooled GbS data to identify markers tightly linked with the *INF* locus (Additional file 1: Figure S1). Furthermore, we determined the genetic variation at the marker locus using a *H. macrophylla* cultivar collection and identified marker sequences which are coupled with mophead and lacecap alleles.

## Results

### A F_1_ population with diploid and triploid individuals segregates for the inflorescence type

In order to develop markers, which are coupled with the *INF* locus, we used a F_1_ population consisting of 422 plants. This population was derived from a cross between a mophead and a lacecap cultivar of *H. macrophylla* ssp. *macrophylla* (Fig. 1a, b). In total, 279 F_1_ plants developed lacecap and 73 F_1_ plants mophead inflorescences, whereas 3 F_1_ plants produced intermediate inflorescence types. In contrast, 67 F_1_ plants were non-flowering in two consecutive seasons (Table 1).

**Table 1:** Numbers of diploid and triploid F_1_ plants that segregated for lacecap and mophead inflorescences

Putative ploidy	Lacecap	Mophead	Intermediate	Non-flowering	In total
Diploid	43	36	0	24	103 (24.4%)
Triploid	234	37	3	43	317 (75.1%)
> 54 chromosomes	2	0	0	0	2 (0.5%)
In total	279	73	3	67	422

Plants with 2C DNA contents between 4.1 and 4.5 pg were classified as diploid, between 6.4 and 6.7 pg as triploid. A 2C DNA content of 7.1 and 8.5 pg indicates more than 54 chromosomes

**Fig. 2:**
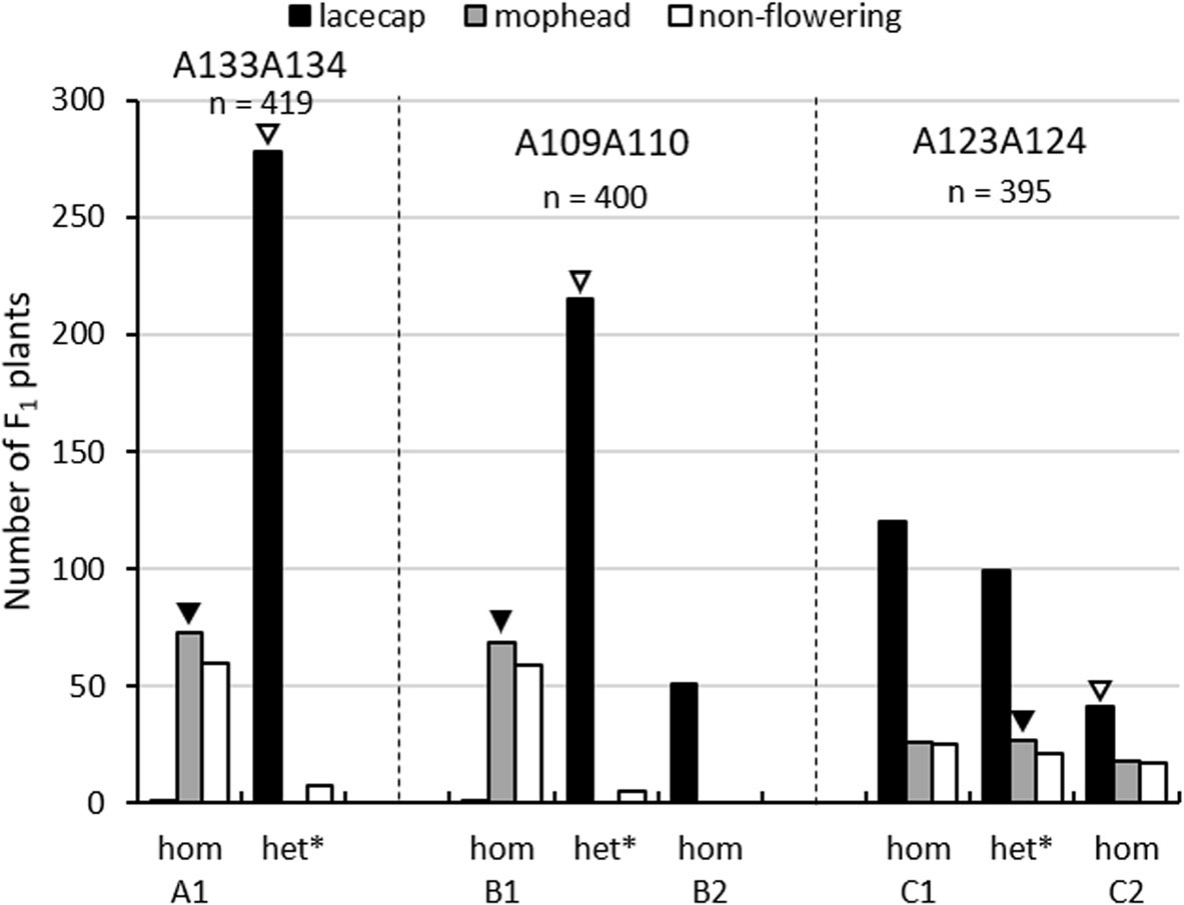
Number of non-flowering, mophead or lacecap F_1_ plants according to their genotype at the marker loci A133A134, A109A110 and A123A124. A1 and B1 are associated with mophead and non-flowering alleles, whereas the alternate alleles A2 and B2 are associated with lacecap and flowering alleles. C1 and C2 are sequence variants of marker A123A124, which segregate independently from the flowering and inflorescence phenotype. Black arrows mark the genotype of the mophead parent, white arrows the genotype of the lacecap parent. ^*^The genotype of heterozygous triploids is unknown; they have either an 1/1/2 or an 1/2/2 genotype

Surprisingly, we found diploid and triploid F_1_ plants, although the mophead and lacecap parent were diploid. Both parents have 36 chromosomes (Fig. 1c, d) and 2C DNA contents of 4.4 and 4.5 pg, respectively. For 103 F_1_ plants, we determined 2C DNA contents between 4.1 and 4.5 pg, on average 4.4 ± 0.95 pg. Thereupon, these plants were classified as diploids. In contrast, 317 F_1_ plants showed 2C DNA contents between 6.4 and 6.7 pg, with an average of 6.6 ± 0.94 pg. These 2C values are typically for triploid *H. macrophylla* cultivars [11], suggesting triploidy for 75.1% of the offspring. Two progenies showed 2C DNA contents of 7.1 and 8.5 pg, respectively, which indicates more than 54 chromosomes assuming aneuploidy or even tetraploidy. All plants of this population showed a normal development. Diploid and triploid plants looked similar and could not be distinguished by eye.

As summarized in Table 1, lacecap inflorescences were produced by 43 diploid, 234 triploid and 2 plants with more than 54 chromosomes. Mophead inflorescences were developed by 36 diploid and 37 triploid plants. Three plants with intermediate inflorescences were triploid. Non-flowering was observed for 24 diploid and 43 triploid plants. Based on these results, the inflorescence and non-flowering phenotypes seem to be independent of the ploidy level. The diploid plants segregated into lacecap and mophead plants in a ratio of 1:1 (Χ^2^ = 0.6202, non-significant at α = 0.05), which was expected for the monogenic, dominant-recessive inheritance of the inflorescence type in a diploid F_1_ population.

### Draft genome assembly of *H. macrophylla* ‘Sir Joseph Banks’

A TruSeq library was generated from genomic DNA of ‘Sir Joseph Banks’ and sequenced on an Illumina NextSeq system. About 147 million 150 bp paired-end reads were generated. These raw sequence data have been deposited at the European Nucleotide Archive (www.ebi.ac.uk) under accession number PRJEB32928. Through de novo sequence assembling, we obtained the assembly L10642. This assembly consisted of 1,519,429 contigs with lengths ranging from 200 to 197,470 nucleotides, yielding in a total sequence length of 1,622,088,866 nucleotides. This assembly has an N50 size of 2,447 nucleotides with 146,645 contigs being this size or larger.

### Bulk analysis using GbS data identifies candidate sequences

The parental plants and 380 F_1_ plants were used for genotyping-by-sequencing. For GbS, we performed restriction site associated (RAD) DNA sequencing, which is a fractional genome-wide sequencing strategy, designed to sequence 0.1 to 10% of a genome. *Msl*I digested, genomic DNA from each sample was used to prepare NGS libraries with 200 to 300 bp DNA fragments, which will yield in joint-end reads with average lengths of 270 bp after paired-end sequencing. These reads present only genomic sequences close to *Msl*I restriction sites, which are randomly distributed across the genome. For each sample, 1,319 to 2,413,261 150 bp paired-end reads were generated, yielding in 1,243 to 2,241,457, on average 939,767 ± 368,315 high quality read pairs. Originally, we tried to obtain genotypic data of each individual to calculate genetic maps for QTL analyses. However, the calculation of genetic maps failed because of insufficient genotyping data in all samples. Absent or incorrect genotyping occurred randomly in all samples, independently of ploidy, and in all markers, mostly due to missing or low read coverage at GbS marker loci.

In order to increase the reliability of GbS information and to use the data of diploid as well as triploid F_1_ plants, we pooled the sequence data of 99 diploid (mophead, lacecap, non-flowering) and 65 mophead plants (diploid and triploid) and created a diploid and a mophead sequence pool containing 92.1 and 58.1 million read pairs, respectively (Table 2). About 94% reads of each pool mapped to the genome assembly. In total, 603,104 (39.7%) contigs of the genome assembly had 1 to 765,278 read matches in the diploid and 527,059 (34.7%) contigs 1 to 367,469 read matches in the mophead pool. These read matches covered in sum 248.9 million (15.3%) and 196.0 million (12.1%) nucleotides of the genome assembly with 1 to more than 300-fold coverage. The average coverage excluding zero coverage regions was 98.7 ± 596.76 for the diploid and 78.9 ± 388.36 for the mophead pool (Table 2). For variant detection, we considered only positions with 15 to 400-fold coverage in the diploid and 8 to 320-fold coverage in the mophead pool. In total, 1,603,884 and 1,808,537 polymorphic positions were called for these pools with allele frequencies between 18 and 100% corresponding with the parameter settings.

**Table 2:** Summary of bulked GbS data, GbS read processing and variant detection

	Diploid pool	Mophead pool
Description of pooled plants	99 diploid F_1_ plants independent of inflorescence type	65 diploid and triploid mophead F_1_ plants
Total number of high-quality read pairs used for read mapping	92,144,015	58,117,508
Total number of mapped reads	173,568,387 (94.2%)	109,338,937 (94.1%)
Number of contigs with read matches	603,104 (39.7%)	527,059 (34.7%)
Number of reads per scored contig	1 to 765,278	1 to 367,469
Sum of covered nucleotides of the draft genome	248.9 million (15.3%)	196.0 million (12.1%)
Average coverage excluding zero coverage regions (± standard deviation)	98.7 ± 596.76	78.9 ± 388.36
Total number of polymorphic calls with allele frequencies between 18 and 100%	1,603,884	1,808,537
Total number of polymorphic positions with allele frequencies between 25 and 75% indicating heterozygosity	606,317	n.d.

We hypothesized that all cross-specific polymorphisms will be polymorphic in the diploid pool, which includes randomly selected diploid mophead, lacecap and non-flowering plants. In contrast, the mophead pool will be monomorphic at the *INF* locus, if the mophead and lacecap parent carry the same recessive *inf* allele in the homozygous and heterozygous stage, respectively, likely due to the small European breeding pool of *Hydrangea*. In the diploid pool, we detected 606,317 polymorphic positions located on 105,367 contigs filtered for allele frequencies between 25 and 75%. Following, we recorded the allele frequency at these positions in the mophead pool. Finally, we selected those contigs that had minimum 2 polymorphic positions with a mean allele frequency between 25 and 75% in the diploid pool and a mean allele frequency above 95% in the mophead pool. In total, 69 contigs with lengths between 213 and 17,716 nucleotides were detected. Subsequent sequence analysis by eye confirmed 35 contigs with positions that were polymorphic in the diploid and monomorphic in the mophead pool, suggesting a physical location of these contig sequences close to the *INF* locus.

### Marker analysis

Varying read coverage, biased or missing sequencing of some alleles, wrong read mapping due to repetitive genome sequences and masking algorithms in computational variant detection can result in “false-positives”. In order to verify candidate regions selected from bioinformatic analysis, we performed subsequently marker tests for these 35 contigs. Primer pairs, which flanked candidate positions, were designed for 21 out of these 35 contigs based on the read mapping data. The primer design failed for the other 14 contigs due to insufficient sequence information or inadequate primer binding sites. Subsequent marker tests using DNA of the parental plants revealed only 7 primer combinations that produced specific PCR products, which showed polymorphisms in one or both of these parents (Additional file 1: Table S1). The other marker tests resulted either in non-specific PCR products or in PCR products, whose sequence was identical in and between both parental plants. Subsequent sequence analysis showed that 3 out of these 7 markers were homozygous in the mophead and heterozygous in the lacecap parent, whereas 2 markers were heterozygous in the mophead and homozygous in the lacecap parent. Two markers were heterozygous in both of these parents. If the mophead inflorescence type is controlled by the same recessive *inf* allele, then we expected that a marker, which is linked to the *INF* locus, must be homozygous in the mophead and heterozygous in the lacecap parent. This qualification was observed for the markers A103A104, A109A110 and A133A134, whereas the other 4 markers revealed false-positive regions.

Following, we selected the candidate markers A109A110 and A133A134. Marker A133A134 is an insertion-deletion marker, which is detected by agarose gel electrophoresis. Marker A109A110 contains 6 single nucleotide polymorphisms, which are detectable by sanger sequencing. The marker analyses were performed for the total F_1_ population, followed by testing for co-segregation with the inflorescence phenotype. For negative control, we used the marker A123A124, which was heterozygous in the mophead and homozygous in the lacecap parent. The results are shown in Fig. 2. At the marker loci A109A110 and A133A134, all analyzed mophead plants, diploid as well as triploid, and one triploid lacecap plant were homozygous as the mophead parent. The other diploid and triploid lacecap plants carried at each of these marker loci the alternative marker sequence derived from the lacecap parent in the heterozygous or even homozygous stage. Thus, the corresponding genotypes showed 99.7% co-segregation with the inflorescence phenotype, indicating a very close linkage of marker A109A110 and A133A134 with the *INF* locus. For each of these markers, the sequence variants present in the mophead parent were in coupling phase with the mophead allele at the *INF* locus, whereas the alternative sequence variants of these markers, which were derived from the lacecap parent, were in coupling phase with the lacecap allele. At the non-candidate marker locus A123A124, the genotypes explained only 49.9% of the inflorescence phenotypes, indicating an independent segregation between marker A123A124 and the *INF* locus as expected.

One out of 3 triploid plants with an intermediate inflorescence type carried the mophead-coupled sequence variants of the markers A133A134 and A109A110 in the homozygous stage, whereas the other 2 plants were heterozygous at both of these loci. The 2 lacecap progenies with more than 54 chromosomes carried at least one of the lacecap-coupled sequence variants at these marker loci.

The marker loci A133A134 and A109A110 are located on different contigs of genome assembly L10642. These contigs have a length of 667 and 5,295 nucleotides, respectively (Additional file 1: Table S1). Both contigs show no overlapping sequences to each other or with other contigs of the genome assembly. Thus, the order of these marker loci and the *INF* locus remains unknown. Furthermore, no genes could be annotated on these contigs, most likely due to the shortness of their sequence. Thus, a further characterization of the *INF* locus was not possible.

Interestingly, 89.6 and 92.2% non-flowering plants were homozygous for the mophead-coupled sequence variants of A133A134 and A109A110 (Fig. 2). Thus, we assume that the non-flowering phenotype is genetically controlled by a major locus, which is linked to the *INF* locus, and that the mophead and non-flowering alleles are in coupling phase.

### 2 out of 6 sequence variants at marker locus A109A110 are associated with the mophead phenotype

In order to analyze the genetic variation at marker locus A109A110, we performed a marker analysis using DNA of 55 varieties of *H. macrophylla*. Previously, 43 out of these varieties were characterized by SSR marker analysis and flowcytometry [11]. Twelve additional cultivars were characterized for their genetic fingerprint and 2C DNA content in this study (Additional file 1: Table S2). Based on these characterizations, our collection included 26 mophead and 29 lacecap genotypes with 2C DNA contents between 4.0 and 8.9 pg (Table 3), revealing diploid, triploid as well as tetraploid genotypes. Furthermore, we had the genomic and phenotypic information about cultivar ‘Sir Joseph Banks’.

**Table 3:** Genotype of 56 *H. macrophylla* varieties at marker locus A109A110

Genotype at marker locus A109A110	*H. macrophylla* varieties
Homozygous for B1 (*n* = 19)	Baby Blue (G075_M_4.4 pg) Bela (G002_M_6.5 pg) Benelux (G003_M_8.9 pg) Bodensee II (G009_M_4.4 pg) Choco Bleu (G068_M_4.5 pg) Diva fiore (G077_M_4.6 pg) Glärnisch (G027_M_4.4 pg) Nikko Blue I (G048_M_4.5 pg) Paris Rampp (G070_M_4.4 pg) Pfau II (G052_M_4.4 pg) R. F. Felton (G079_M_7.0 pg) Renate Steiniger I (G053_M_4.4 pg) Tödi (G057_M_4.5 pg) unknown 1 (G010_M_4.4 pg) unknown 3 (G015_M_6.6 pg) unknown 4 (G016_M_6.6 pg) unknown 7 (G021_M_4.4 pg) unknown 8 (G024_M_4.5 pg) unknown 12 (G044_M_6.7 pg)
Homozygous for B2 (*n* = 10)	Early Blue (G076_M) Forever Pink (G073_M_4.6 pg) Hörnli (G030_M_4.5 pg) Madame Emile Mouillère (G040_M_4.5 pg) Marisii Perfecta (G035_L_4.5 pg) Mathilde Gütges I (G038_M_4.4 pg) Sheila (G071_L_4.4 pg) Sir Joseph Banks (M)/draft genome unknown 2 (G014_M_4.4 pg) unknown 5 (G017_M_6.7 pg)
Homozygous for B3 (*n* = 6)	Bergfink (G004_L_6.7 pg) Buchfink (G005_L_6.7 pg) Buntspecht (G011_L_4.4 pg) Rotschwanz I (G055_L_4.5 pg) Rotschwanz II (G056_L_4.4 pg) Zeisig (G061_L_4.4 pg)
Homozygous for B4 (*n* = 3)	Bachstelze (G001_L_4.3 pg) Gimpel (G026_L_4.4 pg) Libelle (G033_L_4.4 pg)
Homozygous for B5 (*n* = 2)	Mariesii Lilacina (G037_L_4.2 pg) Nikko Blue II (G049_L_4.0 pg)
Homozygous for B6 (*n* = 2)	Mariesii Grandiflora (G036_L_4.3 pg) Veitchii (G059_L_4.2 pg)
Heterozygous for B1 and B3 (*n* = 9)	Blaukehlchen (G006_L_4.5 pg) Blaumeise (G002_L_6.5 pg) Dark Angel (G072_L_4.5 pg) Eisvogel (G012_L_6.6 pg) Grasmücke (G028_L_4.4 pg) Little Prince (G069_L_4.4 pg) Möwe (G041_L_6.6 pg) Nachtigall (G046_L_6.5 pg) Rotkehlchen (G047_L_4.5 pg)
Heterozygous for B1 and B4 (*n* = 4)	Bläuling (G007_L_4.5 pg) Geoffrey Chadbund (G025_L_4.4 pg) Mak20 (G074_L) Sweet Dreams (G078_L_4.5 pg)
Heterozygous for B3 and B4 (*n* = 1)	Zaunkönig (G060_L_4.4 pg)

These varieties were previously characterized for their SSR marker fingerprint (G), inflorescence type (M mophead, L lacecap) and 2C DNA content. 2C DNA contents from 4.0 to 4.6 pg suggest diploidy, from 6.5 to 7.0 pg triploidy, 8.9 pg tetraploidy. Unknown varieties and Roman numerals refer to previous cultivar characterizations according to Hempel et al. [11]

**Fig. 3:**
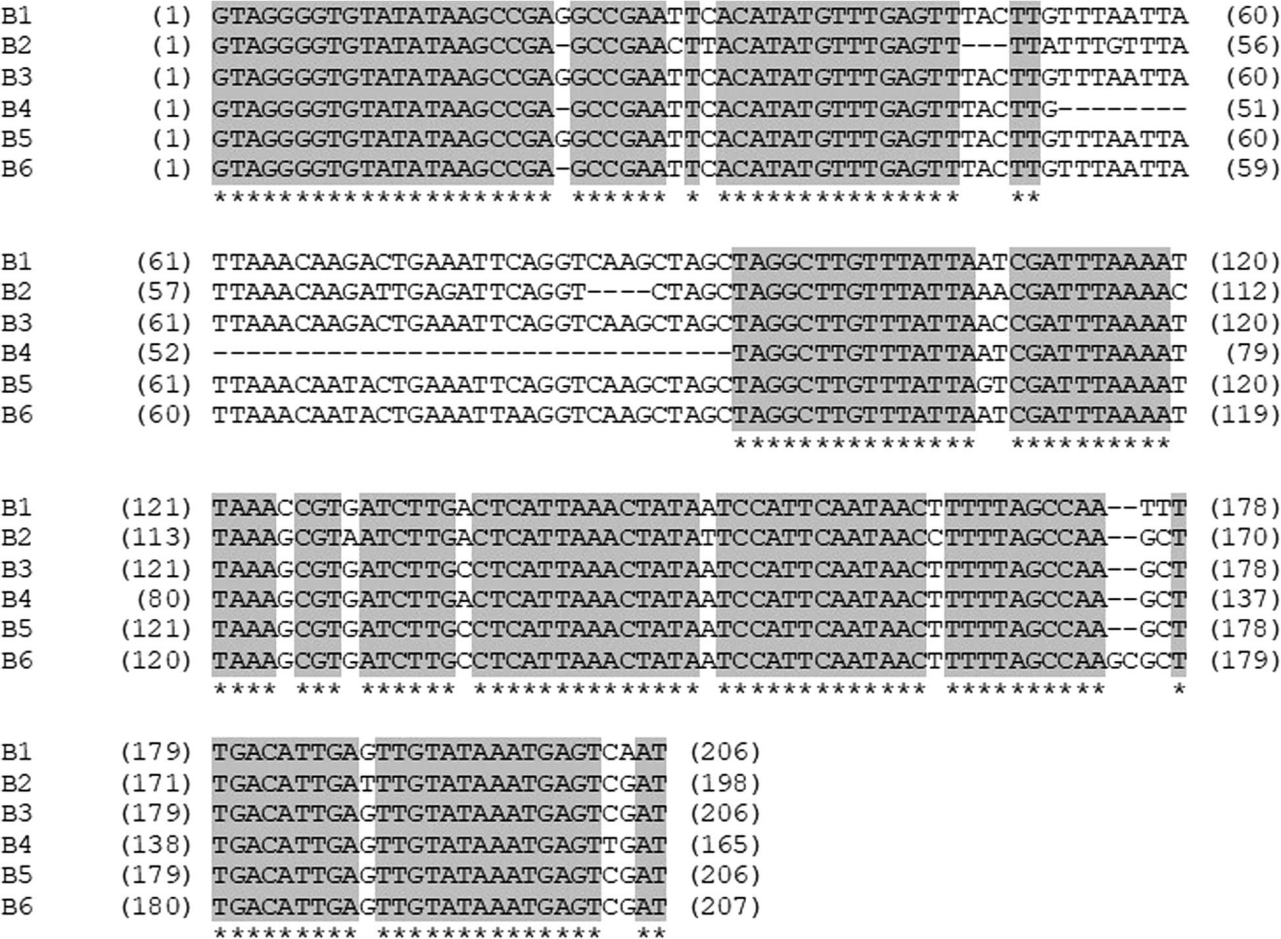
Sequence variants B1–6 at marker locus A109A110. B1 and B2 are associated with the mophead phenotype. B2 is identical with the sequence at contig16908 of the draft genome assembly derived from *H. macrophylla* cultivar ‘Sir Joseph Banks’. Conserved regions are shown in grey

By analyzing this collection, we identified 6 sequence variants at marker locus A109A110 (Fig. 3). These variants differ in several single nucleotides, insertions and deletions, assuming that this marker locus is located in a non-conserved, non-coding region. As shown in Table 3, all mophead varieties carry either the sequence variant B1 or B2 in the homozygous stage. However, 2 out of 10 varieties that are homozygous for B2 develop lacecap inflorescences. The other lacecap varieties are homozygous for the sequence variants B3, B4, B5 or B6 or carry at least one of these variants. Thus, the sequence variants B1 and partially B2 are coupled with mophead alleles at the *INF* locus, whereas the sequence variants B3, B4, B5 and B6 are coupled with dominantly acting lacecap alleles. The partial linkage of B2 with the mophead allele might indicate a subsequent mutation at the *INF* locus or a former cross-over event between marker and *INF* locus.

The triploid lacecap cultivar ‘Blaumeise’ carries heterozygously the sequence variants B1 and B3, while its somatic mophead mutant ‘Bela’ [11, 15] is homozygous for the mophead-coupled B1 variant. Thus, the mutation resulting in ‘Bela’ might be based on a loss of a DNA segment that contained the B3 sequence and fully or partially the coupled lacecap allele, highlighting the tight linkage of marker locus A109A110 and the *INF* locus.

## Discussion

In this study, we have developed a pipeline for rapid marker detection in *H. macrophylla* using partial next generation sequencing data of diploid and triploid F_1_ plants. This pipeline allowed the straight identification of markers tightly linked with the *INF* locus. For *INF* marker A109A110, we detected 6 sequence variants within a collection of 56 *H. macrophylla* varieties. Two out of these sequence variants are coupled with mophead alleles.

Genotyping-by-sequencing is an innovative method for genotyping and mapping [14]. However, the calculation of genetic maps for subsequent linkage analysis of the inflorescence type failed. One reason was the insufficient coverage of GbS loci. The GbS procedure used in this study yielded in many absent or incorrect genotypic data randomly distributed across all samples and markers. Deeper sequencing will certainly promote the accuracy of marker data, but will increase also costs. Frequently, we also observed wrong read mappings due to highly repetitive sequences in the genome of *H. macrophylla*. Wrong mappings result also in false genotypic data. Accurate genotyping is essential for linkage analysis, but seems to be challenging with next generation sequencing methods like GbS.

The calculation of genetic maps is mostly based on diploid offspring. However, only 103 F_1_ plants of our population were diploid, whereas 317 F_1_ plants were triploid. This finding was unexpected because both of the parental plants were diploid. Crossing these plants by hand, makes pollen contaminations unlikely. Recently, Alexander [10] reported the production of triploid F_1_ progenies from a cross between the diploid *H. macrophylla* cultivars ‘Princess Juliana’ and ‘Trophee’. Triploid F_1_ plants were obtained when ‘Trophee’ was used as male parent, whereas the reciprocal cross resulted only in diploid offspring. Alexander [10] assumed spontaneous autopolyploidy via unreduced pollen. This assumption was supported by the bimodal pollen size distribution of ‘Trophee’, which probably indicate reduced and unreduced male gametes [7].

Flow cytometric analysis of pollen showed that the paternal lacecap parent produces unreduced pollen, whereas the female parent developed reduced pollen (data not shown). Although we do not know the quality of female gametes in these parents, our finding suggests that triploid F_1_ plants were most likely derived from sexual autopolyploidization induced by the male parent via 2n gametes. We assume that the production of unreduced gametes in *H. macrophylla* is genotype-specific, which suggests a genetically controlled mechanism. Many of the existing triploid *Hydrangea* cultivars might have resulted from this mechanism. As reviewed by De Storme and Geelen [16], several cytological mechanisms result in 2n gamete formation. These mechanisms involve pre- and post-meiotic genome doubling as well as meiotic restitution. Pre-meiotic endomitosis, endoreduplication or nuclear fusion produces tetraploid meiocytes, which can give diploid gametes after normal meiosis. Or haploid spores perform an extra round of genome duplication in case of post-meiotic genome doubling. Also, meiotic restitution results in formation of 2n gametes. Depending on the restitution type, 2n gametes from a heterozygous plant have either high levels of heterozygosity (first division restitution, FDR) or high levels of homozygosity (second division restitution, SDR). Alternatively, intermediate meiotic restitution (IMR) results in 2n gametes that only partially retain parental heterozygosity at the centromere [16]. Given that the lacecap parent is heterozygous at the *INF* locus, then unreduced gametes contain either the recessive and dominant alleles heterozygously (*INF*/*inf*) or homozygously (*INF*/*INF* or *inf*/*inf*) depending on the cytological mechanisms underlying this 2n gamete formation. Since, we identified 37 triploid mophead F_1_ plants, we might exclude FDR as underlying mechanism. However, the molecular process behind the development of unreduced gametes in *H. macrophylla* is completely unknown. Here, further research is required, because controlled autopolyploidization is highly relevant for breeding of polyploid crops.

Nevertheless, the triploids and GbS data were powerful for bulk sequence analysis, a combination of bulked segregant analysis [17] and mapping-by-sequencing [18]. In both of these methods, normally two bulked DNA samples from a segregating population are used, each bulk with individuals homozygous for alternate alleles of the target region. The comparison of sequence variations between these bulks, mostly based on the subtraction of the relative allele frequencies per position, results in 0 at loci unlinked to the targeted genomic region, but gives the maximum difference of 1 at targeted regions due to the alternate genotypes. However, our F_1_ population structure allowed to create only one bulk containing individuals putatively homozygous for the recessive *inf* allele. An alternating bulk could not be created due to the allelic segregation in backcross populations. Thus, we used only one trait-specific sequence bulk by pooling diploid and triploid F_1_ mophead plants. The diploid pool, which included mophead and lacecap plants, was necessary to distinguish cross specific DNA polymorphisms from DNA polymorphisms specific for the genome assembly of ‘Sir Joseph Banks’. The use of only one trait-specific bulk is sufficient to physically map genetically controlled traits and to identify candidate genes as shown for the *BR*_*1*_ locus of sugar beet [18]. Tränkner et al. [19] used genome-wide sequence data of bulked DNA from only 26 bolting-resistant F_2_ plants and identified a 103 kb interval based on detected recombination sites and 11 candidate genes by subsequent sequence analysis.

Through bulk sequence analysis, we identified two contigs tightly linked to the *INF* locus. However, the sequence or the size of the *INF* locus interval remains unknown. The physical mapping of the *INF* locus was impeded by the strongly fragmented genome assembly as well as the partial genomic data derived from GbS. An annotated reference genome of *H. macrophylla* is currently missing. Thus, we generated an own genome assembly. The quality of our assembly is comparable with another de novo genome assembly derived from *H. macrophylla* ‘Kirakiraboshi’ [13], consisting of many short contigs, whose linkage and order are unknown. The strong fragmentation is most likely due to highly repetitive sequences in the 2.2 Gbp genome of *H. macrophylla*. On the other hand, the GbS method produced only partial sequence data and many contigs had no informative value, such as contigs without read matches or without polymorphic read clusters. Furthermore, the length of read clusters was limited according to the lengths of mapped reads and most of the contig sequences were uncovered. Thus, we could not detect a continuous, homogeneous region for the *INF* locus. Nevertheless, we identified tightly linked markers with 99.7% co-segregation based on about 400 F_1_ plants. Recently, Waki et al. [13] mapped the *INF* locus to linkage group 4 and identified the flanking markers HS527 and HS071. These markers are located 11.7 cM upstream and 5.1 cM downstream of the *INF* locus and explain 93.5 and 96.3% of the inflorescence phenotype based on 351 F_2_ plants, respectively [13]. Our *INF* markers seem to be located closer to the *INF* locus, although these percentages are not really comparable due to the different population types. Our study is based on a heterogenous F_1_ population containing diploid and triploid plants. Cross-over events might be masked in these triploids. A marker analysis using the *INF* markers developed in our study and the F_2_ population described by Waki et al. [13] would allow to confirm these markers with closest linkage to the *INF* locus.

Based on 55 *H. macrophylla* varieties, we identified 6 sequence variants at marker locus A109A110. Two variants are coupled with the mophead allele at the *INF* locus. Nevertheless, the number of alleles at the *INF* locus is unknown. Several hundred *H. macrophylla* cultivars were breed in the twentieth century based on 7 or 8 different plants that were brought to Europe in the 18th and nineteenth century [20]. According to this breeding history, the gene pool used in European breeding programs is rather small. However, 6 sequence variants at a locus near to the breeding-targeted *INF* locus is a rather high number of alleles available in the gene pool. Thus, our study reveals a stronger genetic variation at the *INF* locus of *Hydrangea* cultivars than it was expected from the breeding history.

## Conclusions

We identified two co-dominant markers for the mophead/lacecap inflorescence type through a rapid bulk sequence analysis using diploid and triploid F_1_ plants from *Hydrangea macrophylla*. The bulk sequence analysis described in this study is a rapid and robust method to identify tightly linked markers. It avoids time-consuming and labor-intensive classical linkage analyses. Our pipeline requires neither extensive genotyping nor the construction of genetic maps, and trait-associated markers are developed not randomly but directly locus-specific. Furthermore, it is suitable for non-model organisms with complex genomes and works even with diploid and polyploid plants. The developed *INF* markers are applicable for marker-assisted selection in *Hydrangea* breeding. In addition, these markers are suitable to determine the genetic variation at the locus-of-interest within the gene pool of *Hydrangea*.

## Methods

### Plant material and phenotyping

In June 2013, a hand-cross was performed between a mophead and a lacecap cultivar of *H. macrophylla* ssp. *macrophylla* as part of a breeding program of the company Kötterheinrich-Hortensienkulturen (Lengerich, Germany). Both parental plants were diploid, showing *2n* = *2x* = 36 chromosomes (Fig. 1). The lacecap cultivar, which was used as pollinizer, is a descendant from a cross between a mophead and a lacecap plant. Due to the recessive inheritance of the mophead trait, we assume at the *INF* locus heterozygosity in the lacecap, but homozygosity in the mophead parent. We expect that all other loci are heterogeneously heterozygous and homozygous in both of these parents and that up to 4 different alleles segregate in the offspring population.

Seeds were harvested from the mophead plant in 2014 and sown onto standard propagation soil. F_1_ seedlings were cultivated in 12 cm pots in a frost-free greenhouse of Kötterheinrich-Hortensienkulturen without additional light supply. At the end of each June, all plants were pruned. The inflorescence type was recorded before pruning in 2016 and 2017 on F_1_ plants that had developed more than 5 shoots. Plants without flowers were classified as nonflowering. A plant was recorded as lacecap when its inflorescences were composed of non-decorative flowers at the center and decorative flowers at the periphery of the inflorescence. A mophead plant was classified when the inflorescences were convex, with decorative flowers also in the center of the inflorescences.

The mophead variety ‘Sir Joseph Banks’, cultivated in the Castle Zuschendorf Botanical Garden of TU Dresden, was used to create a *H. macrophylla* draft genome assembly. ‘Sir Joseph Banks’ is the first *Hydrangea* that was brought to Europe from Asia in the eighteenth century. Thus, it is considered as an ancestor of Europe’s Hortensia breeding. In addition, we used 55 other *H. macrophylla* varieties for marker analysis. All cultivars are listed in Table 3.

### Flow cytometry and chromosome counting

The 2C DNA content was estimated by flow cytometry as described by Hempel et al. [11]. *Pisum sativum* L. ‘Ctirad’ with a 2C DNA content of 9.09 pg was used as internal standard. Leave samples of F_1_ individuals and standard were chopped in 1 ml Galbraith’s buffer (45 mM MgCl_2_, 20 mM MOPS, 30 mM sodium citrate, 0.1% (v/v) Triton X-100, pH 7) freshly supplemented with 50 μg/ml propidium iodide, 50 μg/ml RNAse A and 1% (w/v) PVP 25. The homogenate was passed through a 30 μm CellTrics filter (Partec) and analyzed using a Partec CyFlow Space analyzer with a 488 nm blue solid-state laser at a flow rate of 0.1 μl/s. Data analysis was performed using the software FloMax version 2.70 (Quantum Analysis GmbH). About 10,000 nuclei were analyzed for each sample-standard-mixture. High quality peaks were determined at CV < 4%. The 2C DNA content of each sample was calculated as follows: 2C DNA content sample = mean fluorescence value of sample x 9.09 pg / mean fluorescence value of *P. sativum*.

For chromosome counting, DAPI-stained chromosomes of root tip cells were prepared and counted as described in detail by Hempel et al. [11].

### DNA extraction

Genomic DNA was extracted from 100 mg frozen leaf sample using the DNeasy® Plant Mini Kit (Qiagen). DNA was eluted with double distilled water. DNA amount and quality were determined on 1% agarose gels against λ DNA standards (Thermo Fisher Scientific) and using the NanoDrop 2000c (Thermo Fisher Scientific).

### Genome assembly L10642 of *H. macrophylla* ‘Sir Joseph Banks’

Genomic DNA of *H. macrophylla* ‘Sir Joseph Banks’ (2.2 μg) was used for constructing an amp-free TruSeq library. This library was sequenced on an Illumina NextSeq system yielding in 147 million 150 bp paired-end raw reads with an insert size of 300 bp. The raw data were processed using the software CLC assembly cell beta (version 4.0.6 for Linux). Prior to the assembly, duplicate reads, which were generated during the amplification step of the library preparation, were removed and adapter sequences as well as low quality nucleotides (< Q20) were trimmed off of the read data. The trimmed reads were de novo assembled in CLC in scaffolding mode accounting for the paired-end insert size of 300 bp.

### Genotyping-by-sequencing

Indexed and normalized genotyping-by-sequencing libraries were constructed by LGC Genomics GmbH (Berlin, Germany), using 100–200 ng genomic, *Msl*I digested DNA per sample. These libraries were multiplexed and sequenced with the Illumina NextSeq 500 V2 system. Raw sequencing data were processed by LGC Genomics GmbH (Berlin, Germany), including de-multiplexing, clipping of adapter sequences, restriction enzyme site filtering and quality trimming (minimum average Phred quality score of 20 over a window of 10 nucleotides, minimum read length of 20 nucleotides).

### GbS data processing for bulk sequence analysis

Quality trimmed GbS reads were subsequently processed using the software CLC Genomics Workbench 10.1.1. Paired-end reads of each sample were imported with a paired-end distance of 1–1000 nucleotides. Then, the paired-end reads of 99 diploid plants (lacecap, mophead and non-flowering) and 65 mophead plants (diploid and triploid) were combined, in order to create a diploid and a mophead sequence pool. Both sequence pools were separately mapped against the draft genome assembly with mismatch cost 2, insertion and deletion cost 1, length fraction 0.5, and similarity fraction 0.9. Only uniquely mapped paired-end and single reads were considered in subsequent steps, allowing duplicates. The average coverage excluding zero coverage regions was determined for each sequence pool using the CLC Create-Detailed-Mapping-Report function. Sequence variants were detected using the CLC Basic-Variant-Detection tool with ploidy 4, minimum coverage 15 for the diploid and 8 for the mophead pool, ignoring positions with more than 4-fold coverage compared to the corresponding average coverage (excluding zero coverage regions), minimum count 3, and minimum frequency 18%.

Polymorphic positions with allele frequencies between 25 and 75% were determined for the diploid pool and compared with the allele frequency at the same position in the mophead pool using in-house macro scripts in Excel. We considered a SNP or InDel position as candidate if it was polymorphic in the diploid pool and monomorphic in the mophead pool. The first condition is necessary to exclude sequence polymorphisms due to differences to the draft genome assembly derived from ‘Sir Joseph Banks’. The second condition allows to identify DNA sequences coupled with mophead alleles.

### Primer design and marker analysis

Molecular markers were developed based on pooled GbS read mapping data. Primers flanking candidate positions were designed using the software tool OligoCal [21]. All primers were obtained from Metabion International AG. PCR assays were done in a total volume of 25 μl containing 1x PCR buffer including MgCl_2_, 0.2 mM dNTP mix, 0.2 mM forward and reverse primer, 0.02 mM U mi-Taq DNA polymerase (Metabion International AG) and up to 10 ng DNA. PCR products were separated by agarose gel electrophoresis or Sanger sequenced (GATC - Eurofins Genomics). All markers including the primer sequences and PCR conditions are listed in Additional file 1: Table S1.

## Additional file

Additional file 1: **Table S1.** Marker list. **Table S2.** 2C DNA content, inflorescence type and SSR marker fingerprint of 12 *H. macrophylla* cultivars. **Figure S1.** Pipeline for *INF* marker development using RAD GbS data of diploid and triploid F_1_ plants. (DOCX 91 kb)

## Data Availability

Raw sequence data of the draft genome assembly have been deposited at the European Nucleotide Archive (www.ebi.ac.uk) under accession number PRJEB32928. GbS data can be requested from the authors. Unreleased breeding material of the company Kötterheinrich-Hortensienkulturen was used in this study. This material can be requested under restrictions from the company.

## Abbreviations

GbS: Genotyping-by-sequencing
InDel: Insert-deletion polymorphism
*INF*: *INFLORESCENCE TYPE* locus
SNP: Single nucleotide polymorphism
