# Supplementary material for: Rapid identification of inflorescence type markers by genotyping-by-sequencing of diploid and triploid F1 plants of Hydrangea macrophylla
Source: BMC Genet. 2019 Jul 23;20:60. doi: 10.1186/s12863-019-0764-6 (PMC6651981; doi:10.1186/s12863-019-0764-6)
Supplement: Supplementary file 1 — Table S1. Marker list. Table S2. 2C DNA content, inflorescence type and SSR marker fingerprint of 12 H. macrophylla cultivars. Figure S1. Pipeline for INF marker development using RAD GbS data of diploid and triploid F1 plants. (DOCX 91 kb) [file 12863_2019_764_MOESM1_ESM.docx]

## Supplementary

Table S1 Marker list

| Marker | Contig L10642 (contig length) | Forward primer  5‘🡪 3‘ | Reverse primer  5‘ 🡪 3‘ | Marker detection | Length of PCR product in bp according to L10642 | Polymorphisms mophead parent | Polymorphisms lacecap parent | Linkage with the *INF* locus |
| --- | --- | --- | --- | --- | --- | --- | --- | --- |
| A099A100 | 276104  (12,539 nt) | A099: CACGAGAGC  ATTGATAGTACTG | A100: GTAAGTTTAAT  TRAGAAAAAGACTCG | PCR at 94°C, 3' + [(94°, 30'' + 60°C, 30'' + 72°C, 30'') x 32] + 72°C, 5';  sequencing with A100 | 118 | A099:  23 A/G  48 T/C | A099:  23 A/G  48 T/C | n.d. |
| A103A104 | 1152358  (248 nt) | A103: GTTGTACACA  TTGACCAATTAC | A104: CAACAAGAAA  TGCTTTATGTACAC | PCR at 94°C, 3' + [(94°, 30'' + 56°C, 30'' + 72°C, 30'') x 32] + 72°C, 5';  sequencing with A104 | 227 | A103:  26^27 A/A  27 A/A  34 G/G  100 T/T | A103:  27 A/C  34 G/-  100 T/C  141^142 -/A | n.d. |
| A109A110 | 16908  (5,295 nt) | A109: GTAGGGGTGT  ATATAAGCCGAG | A110: GCTTGATAAATG  GCTTATATACGAAC | PCR at 94°C, 3' + [(94°, 30'' + 62°C, 30'' + 72°C, 30'') x 32] + 72°C, 5';  sequencing with A110 | 272 | A109:  101 T/T  117 C/C  128 A/A  168 T/T  169 T/T  196 A/A | A109:  101 T/C  117 C/G  128 A/C  168 T/G  169 T/C  196 A/G | yes |
| A123A124 | 534403  (2,246 nt) | A123: GTTGCTGAC  CTTTAAATCTTTC | A124: GGAATAGAAA  ACAAAACTATCG | PCR at 94°C, 3' + [(94°, 30'' + 55°C, 30'' + 72°C, 30'') x 32] + 72°C, 5';  sequencing with A124 | 185 | A123:  95 A/T  126 G/A  135 A/T | A123:  95 A/A  126 G/G  135 A/A | no |
| A125A126 | 229528  (2,205 nt) | A125: GTAGGTTTGC  CGTCACTTAG | A126: CTTAGACTGTA  AAATACAATTC | PCR at 94°C, 3' + [(94°, 30'' + 55°C, 30'' + 72°C, 30'') x 32] + 72°C, 5';  sequencing with A125 | 105 | A126:  23 C/T  24 G/A  50 A/G | A126:  23 C/T  24 G/A  50 A/G | n.d. |
| A129A130 | 1146015  (1,404 nt) | A129: GTGCTGGGTT  GCTCCTTGATG | A130: CGATAGAGGG  TGGATAATCCTG | PCR at 94°C, 3' + [(94°, 30'' + 62°C, 30'' + 72°C, 30'') x 32] + 72°C, 5';  sequencing with A129 | 128 | A130:  33 G/A  76 T/C | A130:  33 G/G  76 T/T | n.d. |
| A133A134 | 1287540  (667 nt) | A133: CTCAACTTCA  CTAGATCTTGC | A134: GATAGTACAGT  GATATTGGTG | PCR at 94°C, 3' + [(94°, 30'' + 55°C, 30'' + 72°C, 30'') x 32] + 72°C, 5';  2% agarose gel (sequencing with A133) | 173 | 159 bp PCR fragment  A134:  24 -/-  44 T/T  45 A/A  68 T/T  70 T/T  77 -/-  85 G/G  97 -/-  99^100 G/G  133^145 12 bp deletion | 173+159bp PCR fragment  A134:  24 -/G  44 T/G  45 A/G  68 T/C  70 T/G  77 -/G  85 G/A  97 -/C  99^100 G/-  133^145  -/ATTGGGGG  GGTT | yes |

Table S2 2C DNA content, inflorescence type and SSR marker fingerprint of 12 *H. macrophylla* cultivars

| Cultivar | 2C DNA content [pg] | Inflorescence type | Binary index matrix of SSR markers*  #1__#2___#3___#4_____#5____#6_____#7____#8______#9_______#10______#11_____#12 | SSR fingerprint ID** |
| --- | --- | --- | --- | --- |
| Choco Bleu | 4.50 | mophead | 0001_10_00001_00001_10100_01001_1000010_00001_10000100_000001000_0110000_001100000 | G068 |
| Little Prince | 4.37 | lacecap | 0001_10_00001_00101_00001_01100_0000010_00001_00100000_001001000_0100001_100100000 | G069 |
| Paris | 4.42 | mophead | 0001_10_00001_00101_10000_01100_0010010_00001_00100000_001001000_0100000_000100001 | G070 |
| Sheila | 4.43 | lacecap | 0001_11_00001_00100_10000_01000_0000010_00001_01100000_000011000_0010001_000100000 | G071 |
| Dark Angel | 4.48 | lacecap | 0001_11_00001_00100_10001_01100_0000010_00001_00100000_001010000_0100001_000100001 | G072 |
| Forever Pink | 4.61 | mophead | 0001_11_00001_00101_10100_01000_0010010_uuuuu_10100000_000001000_0110000_001000001 | G073 |
| Mak20 | n.d. | lacecap | 0001_11_00011_00011_10000_00101_0000010_00101_00100001_000100001_0010010_000000101 | G074 |
| Baby Blue | 4.44 | mophead | 0101_10_00001_00001_10001_01000_0010010_10001_00100000_000001000_0100000_000100000 | G075 |
| Early Blue | n.d. | mophead | 0101_10_00001_00001_10001_01000_0010010_10001_00100000_000001000_0100000_000100000 | G076 |
| Diva fiore | 4.55 | mophead | 0101_11_00001_00001_10000_01001_0010010_00001_00100100_000001100_0110000_000000001 | G077 |
| Sweet Dreams | 4.48 | lacecap | 0101_11_00001_00100_00101_00100_0000010_00001_00100000_000001000_0011000_000100000 | G078 |
| R.F. Felton | 6.97 | mophead | 0101_11_10001_00001_10100_01001_0100010_00001_10100000_000001000_0011000_000100001 | G079 |

* Markers according to Hempel et al. [11], allele-specific PCR fragment lengths for marker #1: 155/146/143/140 bp, for marker #2: 167/161 bp, for marker #3: 162/159/153/150/147 bp, for marker #4: 186/180/174/170/168 bp, for marker #5: 155/152/149/148/146 bp, for marker #6: 156/153/150/147/138 bp, for marker #7: 153/150/147/143/139/137/134 bp, for marker #8: 158/147/144/141/138 bp, for marker #9: 150/147/143/140/138/135/130/124 bp, for marker #10: 180/177/172/167/161/152/143/134/128 bp, for marker #11: 135/132/129/126/123/120/118 bp, and for marker #12: 126/123/120/117/113/110/107/104/101 bp

** continued according to Hempel et al. [11]


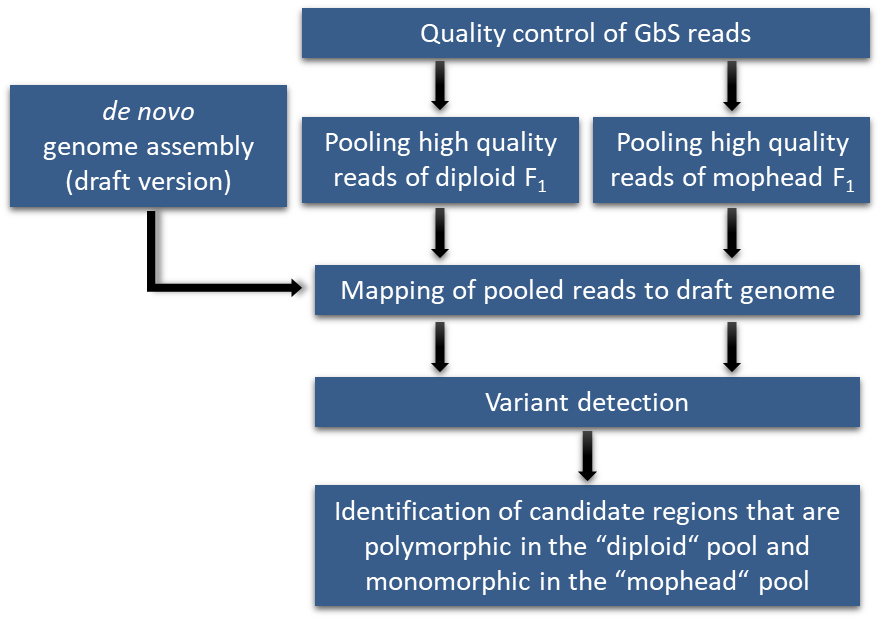


Figure S1 Pipeline for *INF* marker development using RAD GbS data of diploid and triploid F_1_ plants
